# So cute, I could wait: the effect of cuteness on consumer patience

**DOI:** 10.3389/fpsyg.2024.1380505

**Published:** 2024-05-13

**Authors:** Xiaoran Wang, Jing Jiang, Xiadan Zhang

**Affiliations:** ^1^School of Business, Renmin University of China, Beijing, China; ^2^School of Journalism & Communication, Jinan University, Guangzhou, China

**Keywords:** cuteness, consumer patience, perceived social support, time pressure, word-of-mouth positivity

## Abstract

While waiting has been a prevalent and mentally taxing experience for consumers in marketing scenarios, little research has explored situational factors that enhance consumer patience. Drawing on the priming theory, attachment theory, and conservation of resources theory, the current research examines how cuteness as a situational factor affects consumer patience. Across five experiments (*N* = 1030), we demonstrate that exposure to cuteness enhances consumer patience (Study 1). Moreover, we uncover that the effect is driven by perceived social support employing both mediation (Study 2) and moderation approaches (Study 3). Furthermore, we identify time pressure as the moderator, such that the effect of cuteness on consumer patience only exists among individuals under low time pressure and disappears for those under high time pressure (Study 4). Finally, we examine the downstream consequence of consumer patience for word-of-mouth positivity (Study 5). These findings contribute to the literature on cuteness, patience, and perceived social support, while also offering practical implications for companies seeking to enhance consumer patience.

## 1 Introduction

Cuteness, characterized by the presence of infantile features (Lorenz, 1970), is a prevalent element in marketing to increase brand recognition and product evaluation (Belch and Belch, 2004). It has been employed in numerous marketing materials, such as mascots and advertisements. For instance, *Geico*’s Gecko, one of the most recognizable and beloved advertising mascots, has apparent babylike features, including a bulging forehead, a small body, and large eyes. The advertisements of *Coca-Cola* featuring adorable polar bears have also become a favored symbol of their marketing campaigns. Despite the prevalence and success of cuteness in marketing, research on its effects on specific consumer behavior is worth digging deeper. Recently, cuteness has been employed in waiting scenarios. A Korean coffee chain, *Mann Coffee*, utilizes cute teddy bears as table trackers with which customers wait to be served after placing their orders. Inspired by this novel application of cuteness in marketing, our research endeavors to investigate whether and how exposure to cuteness increases consumer patience.

Waiting is a mentally taxing experience that almost everyone has gone through (Dai and Fishbach, 2013), from waiting for a table in a restaurant to lining up to enter a retail store. According to a survey conducted by Waitwhile (2018), 64% of Americans have to wait in line multiple times per week, with 44% expressing reduced satisfaction with businesses that experience lines. Similarly, in the UK, nearly two-thirds of people concur that society, nowadays, is generally less patient than it was a decade ago (Hughes, 2023). As a consequence, enhancing consumer patience in waiting scenarios has important practical significance. Accordingly, abundant research has examined factors that influence consumer patience, involving individual differences (Thompson et al., 2020), perceptual factors (Bartels and Urminsky, 2011; Romero et al., 2019), emotions (Pyone and Isen, 2011; Huang et al., 2016), and situational factors (Garaus and Wagner, 2019; Kim and Zauberman, 2019; Shaddy and Lee, 2020). Despite the fact that situational factors can be easily applied in marketing practice, there is little research on situational factors that enhance consumer patience. In order to bridge this gap, our research proposes cuteness as a novel situational factor to increase consumer patience.

Prior research on cuteness has mainly concentrated on its care-releasing functions, demonstrating that cuteness elicits parenting motivation and caretaking behavior (Glocker et al., 2009; Li and Yan, 2021). However, our research takes a unique perspective by emphasizing the social functions of cuteness. In light of the priming theory and attachment theory, we put forward that exposure to cuteness enhances consumers’ perceptions of social support. Furthermore, we suggest that perceived social support facilitates consumers’ coping with psychological threats and time loss during waiting, ultimately resulting in increased consumer patience. Additionally, the current research examines the moderating role of time pressure, indicating that the effect of cuteness on consumer patience only exists in situations of low time pressure but is diminished for consumers experiencing high time pressure.

The current research provides several notable contributions to both theoretical understanding and marketing practice. Firstly, by exploring the social functions of cuteness, our findings expand the existing body of research on cuteness. Secondly, we extend the literature on consumer patience by recognizing cuteness as a novel situational determinant and its positive impact on consumer patience. Thirdly, our research enriches the functions of perceived social support, as it can counteract the psychological threats and time loss associated with waiting. Moreover, we pinpoint time pressure as a moderating factor, revealing that the cuteness effect is malleable. Finally, our findings indicate that incorporating cute elements into waiting scenarios increases perceived social support, enhancing consumer patience and ultimately fostering word-of-mouth positivity.

## 2 Theoretical background and hypotheses

### 2.1 Determinants of consumer patience

Waiting is mentally taxing and generally considered a negative experience (Dai and Fishbach, 2013; Sun et al., 2022). As the pain of spending derives from the feeling of losing money (Rick et al., 2008), the pain of waiting may also be associated with the loss of time, as both money and time are valuable resources for individuals (Mogilner, 2010). Nevertheless, waiting is ubiquitous in the consumption process, such as waiting for a gift from retailers, waiting to be seated in restaurants, and waiting to enter a retail store, which requires a great deal of consumer patience. Consumer patience, defined as the willingness to wait for a desirable product or service when facing a delay (Thompson et al., 2020), contributes to one’s psychological well-being (Schnitker et al., 2017) and evaluation of the service providers (Djelassi et al., 2018). Given its importance for both individuals and companies, numerous determinants of patience have been explored in the past decades.

Existing literature has identified four categories of determinants that influence consumer patience (see Table 1), including individual differences (Thompson et al., 2020), perceptual factors (Bartels and Urminsky, 2011; Romero et al., 2019), emotions (Huang et al., 2016), and situational factors (Kim and Zauberman, 2019; Shaddy and Lee, 2020). Firstly, regarding individual differences, people who experienced low socioeconomic status during their childhood are more prone to exert self-control by waiting for a chosen alternative, as opposed to trying to control their environment (Thompson et al., 2020). In contrast to individuals from Eastern cultures, westerners tend to be less patient and value immediate consumption (Zhang and Shrum, 2009). Secondly, pertaining to perceptual factors, time perception plays a vital role in consumer patience due to the valuable resource (i.e., time) consumed during waiting. Prior studies have shown that spatial representation of time (Romero et al., 2019), time units (Siddiqui et al., 2018), and future event markers within a duration (May, 2017) can alter one’s time perception and consequently influence their patience. Additionally, self-perception, such as self-continuity, can also impact patience. When individuals perceive a close connection between their present self and their future self, they tend to wait patiently so that their future self can receive larger benefits (Bartels and Urminsky, 2011). Thirdly, positive mood can also bring about a future-oriented time perspective by enhancing cognitive flexibility and construal level (Pyone and Isen, 2011). Emotions such as nostalgia increase consumer patience by inducing a savoring mindset (Huang et al., 2016).

**Table 1:** Summary of prior research on determinants of patience.

Studies	Categories of determinants	Subcategories of determinants	Determinants	Key findings
Curry et al. (2008)	Individual differences	Trait	Cooperativeness	One’s cooperativeness is positively correlated with patience.
Zhang and Shrum (2009)		Cultural	Western (vs. Eastern) cultures	Individuals from Western (vs. Eastern) cultures tend to be less patient and value immediate consumption.
Thompson et al. (2020)		Demographic	Childhood socioeconomic status	People with low (vs. high) childhood socioeconomic status are more likely to wait for a desired alternative.
Bartels and Urminsky (2011)	Perceptual factors	Self-perception	Self-continuity	When individuals perceive their present selves as closely connected to their future selves, they are more willing to wait patiently.
May and Monga (2014)		Time perception	Anthropomorphism of time	Anthropomorphism of time decreases patience for powerless individuals.
May (2017)			Future event markers within a time duration	For individuals relying on emotions (vs. reason), the number of events within a duration increases (vs. decreases) patience.
Siddiqui et al. (2018)			Time units	Large time units (e.g., 2 days) lead to greater patience when rewards are hedonic compared to small time units (e.g., 48 h).
Romero et al. (2019)			Spatial representation of time	When individuals perceive a close connection between their present self and their future self, they tend to wait patiently.
Pyone and Isen (2011)	Emotions	Emotional valence	Positive mood	Positive mood enhances patience by inducing a future-oriented time perspective.
Lempert et al. (2016)		Emotional arousal	Arousal	Increased emotional arousal caused by reward concreteness brings about impatience.
Huang et al. (2016)		Specific emotions	Nostalgia	Feelings of nostalgia increases consumer patience.
Zhong & DeVoe (2010)	Situational factors	Factors that have negative effects on patience	Exposure to fast food	Incidental exposure to fast food induces consumer impatience.
Kim and Zauberman (2013)			Exposure to sexual cues	Exposure to sexual cues results in greater impatience.
Kim and Zauberman (2019)			Music tempo	Fast tempo music leads to impatience.
Shaddy and Lee (2020)			Exposure to price promotions	Exposure to price promotions decreases patience.
Garaus and Wagner (2019)		Factors that have positive effects on patience	Retail environment distracters	Digital signage makes consumers perceive less waiting time.
The current research			Exposure to cuteness cues	Exposure to cuteness increases consumer patience.

Lastly, many situational factors (i.e., external circumstances, context, or environmental conditions) have been found to cause consumer impatience, such as exposure to sexual cues (Kim and Zauberman, 2013), fast tempo music (Kim and Zauberman, 2019), and price promotions (Shaddy and Lee, 2020). Digital signage in retail settings has been found to create a perception of shorter waiting time for consumers, ultimately enhancing their overall store satisfaction (Garaus and Wagner, 2019). Apart from this, minimal research has been undertaken to explore situational factors that increase consumer patience, which is conducive to consumers’ waiting satisfaction and a positive consumption experience (Gui et al., 2021). To address this gap, we innovatively propose exposure to cuteness as a situational factor since it also functions in one’s external physical environment, and examine how it increases consumer patience.

### 2.2 Cuteness and its functions

Cuteness examined in the current research refers to a set of physical attributes found in newborns, including a protruding forehead, sizable eyes, and rounded cheeks (Lorenz, 1970). From an evolutionary perspective, cuteness has commonly been considered an innate releaser of caretaking behaviors toward offspring (Glocker et al., 2009), thereby promoting the survival and reproduction of the human species (Lorenz, 1970; Sherman and Haidt, 2011). Owing to parental instincts, research has demonstrated that the faces of infants effectively attract and hold people’s attention (Brosch et al., 2007), are judged as more likable and attractive (Luo et al., 2011), and lead to behavioral carefulness (Sherman et al., 2009). Previous research has primarily emphasized the function of cuteness in eliciting care and has extended the range of cute agents from human babies to human adults (Gorn et al., 2008), animals (Shin and Mattila, 2021), and even inanimate objects (Nenkov and Scott, 2014).

Based on the care-releasing function of cuteness, prior research has examined people’s emotional and cognitive responses to cuteness and their behavioral consequences (Septianto and Paramita, 2021; Shin and Mattila, 2021; Septianto and Kwon, 2022; Yang et al., 2022). Regarding emotional responses, visual cuteness cues are found to elicit feelings of tenderness and thus prompt prosocial and sustainable behavior when the beneficiaries are related to the source of cuteness (Wang et al., 2017). Additionally, incidental exposure to cuteness cues evokes kama muta, a feeling of being moved or touched, increasing consumers’ prosocial behavior toward non-cute targets (Shin and Mattila, 2021). From a cognitive perspective, the cuteness of brand logos makes consumers perceive the brand as possessing greater growth potential and consequently boosts brand attitude among those feeling hopeful (Septianto and Paramita, 2021). Furthermore, a logo with a cute design enhances consumers’ motivation to safeguard the brand from harm, resulting in less punishment following a brand transgression (Septianto and Kwon, 2022). Of note, sometimes there is a gender difference in the effect of cuteness due to gender-specific parenting stereotypes (Li et al., 2019). Once caring motivation is triggered by cuteness, men tend to display a preference for risk-seeking behavior, while women generally lean toward risk aversion (Li and Yan, 2021). While the care-releasing functions of cuteness have been well recognized, Sherman and Haidt (2011) proposed that cuteness actually functions as a releaser of sociality. In line with this perspective, the current research is focused on investigating the social functions of cuteness. Specifically, we will investigate how exposure to cuteness influences consumer patience, with perceived social support serving as the underlying mechanism.

### 2.3 Cuteness, perceived social support, and consumer patience

Perceived social support refers to “a feeling of attachment to a person or group that is perceived as caring or loving” (Hobfoll and Stokes, 1988, p. 499). A possible positive correlation between exposure to cuteness and perceptions of social support has been suggested in the literature on theories of priming, attachment theory, developmental psychology, and neuroscience. To begin with, in light of theories of priming, being exposed to situational cues (e.g., cuteness) can trigger related mental representations and associations in an individual’s memory, making the activated concepts more accessible and unconsciously influencing the subsequent perception (Bargh et al., 1986). Given this premise, we propose that exposure to cuteness may activate concepts related to social support, thereby leading to perceptions of social support. The existing literature on attachment theory and developmental psychology has hinted at the associations between cuteness and social support in one’s memory.

In the first place, according to attachment theory, children have an innate desire to regulate and fulfill their attachment needs to feel secure (Bowlby and Holmes, 2012; Baumeister and Leary, 2017). At the time of separation from their caregivers, they often turn to their favored objects as transitional objects to substitute for their attachment figures and manage the separation stress (Bowlby and Holmes, 2012). Since cute agents possess vulnerability (Nenkov and Scott, 2014; Li and Eastman, 2023), they are perceived as non-threatening (i.e., they do not criticize or judge people) and capable of providing unconditional support (Nicholas and Gullone, 2001; Allen, 2003). Consequently, cute agents, such as toys, often become children’s favored objects, serving as transitional objects (Fortuna et al., 2014). In the second, the developmental psychology literature indicates that children have a tendency to anthropomorphize their transitional objects like cute toys (e.g., Curious George) and become strongly attached to them (Gjersoe et al., 2015). This tendency persists throughout adulthood. In the case of adults, cuteness can suddenly arouse a communal sharing relationship (Steinnes et al., 2019; Shin and Mattila, 2021), featuring equivalence, caring, and trust (Fiske et al., 2019). Physical contact with cute inanimate objects (i.e., teddy bears) can even mitigate the detrimental impacts of social exclusion (Tai et al., 2011). Therefore, cuteness has been a significant source of social support since one’s childhood, providing necessary emotional support and comfort to deal with stressful situations (Nicholas and Gullone, 2001). Drawing from theories of priming, exposure to cuteness may activate concepts such as sharing, support, and comfort, thus increasing perceptions of social support. Finally, neuroscience evidence shows that exposure to cuteness activates brain regions related to both attachment and reward, implying its potential to provide social rewards (Glocker et al., 2009; Kringelbach et al., 2016). Taken together, we suggest that cuteness, a releaser of sociality (Sherman and Haidt, 2011), promotes consumers’ perceptions of social support.

Furthermore, as a fundamental psychological mechanism, social support protects people from adverse experiences, particularly when they feel threatened (Bowlby and Holmes, 2012; Xu et al., 2015). We further propose that perceptions of social support help people better cope with the psychological threats and the loss of time during the wait, leading to greater consumer patience. For one thing, in terms of attachment theory, threatening events automatically activate the attachment behavioral system (Mikulincer et al., 2003; Bowlby and Holmes, 2012). People are motivated to seek social support from others through their innate attachment system when they suffer from painful experiences (Bowlby and Holmes, 2012). Reversely, chronically perceiving social support, such as engaging in high-quality relationships, is beneficial for consumers to cope with stress and buffer pains (Mikulincer et al., 2003). As previously noted, waiting is a mentally costly experience (Dai and Fishbach, 2013; Sun et al., 2022). In such cases, social support may alleviate the psychological threats associated with waiting and thus enhance consumer patience.

For another, according to conservation of resources (COR) theory, there is a resource substitution hypothesis, suggesting that different resources can serve as substitutes for one another in addressing the challenges posed by the (potential) loss of the latter (Hobfoll et al., 1990). Previous research has demonstrated the role of perceived social support in alleviating both physical pain (Zhou and Gao, 2008) and spending pain (Xu et al., 2015), primarily because social support as a psychological resource substitutes for the loss of physical comfort and money, respectively. Considering that energy, money, time, and social support are all valuable resources for individuals (Hobfoll, 1989), we contend that social support may also be able to substitute for the loss of time in the waiting process and therefore enhance consumer patience. Recent research also suggests that consumers shopping with friends (vs. alone), who are assumed to perceive greater social support, demonstrate a greater willingness to wait for a desired product (Gui et al., 2021). In light of the above, we put forward that exposure to cuteness enhances perceived social support among consumers, and subsequently enables them to wait more patiently for their chosen alternative. Specifically, we hypothesize as follows:

*H1*: Exposure to cuteness increases consumer patience.

*H2*: Perceived social support mediates the effect of cuteness on consumer patience.

### 2.4 The moderating role of time pressure

In our research, time pressure refers to the perception of being constrained by the available time to complete a specific task (Iyer, 1989). We posit that time pressure as a situational factor acts a moderating role in the effect of exposure to cuteness on consumer patience from the motivation perspective. Time pressure has been found to enhance one’s levels of arousal, resulting in an increased motivation to complete the given task (Huddleston et al., 2018). In consequence, they may give prominence to efficiency (Song et al., 2023) and prioritize the dominant task over the relatively subtle influence of exposure to cuteness (Dijksterhuis and Bargh, 2001), further inhibiting its effect on consumer patience. Overall, we propose that in the case of consumers experiencing high time pressure, occupied with another task, the effect of cuteness on consumer patience will be mitigated. Conversely, for consumers experiencing low time pressure during the process of waiting, the effect remains. Therefore, we propose the following hypothesis:

*H3*: Time pressure moderates the effect of exposure to cuteness on consumer patience.

### 2.5 Exposure to cuteness enhances word-of-mouth positivity via perceived social support and consumer patience

As waiting becomes increasingly inevitable in consumption scenarios, consumers’ willingness to wait patiently for a chosen alternative (i.e., consumer patience) has substantial downstream outcomes. Consumer patience has been found to be positively associated with the perceived quality of products (Lee and Yoon, 2023), evaluation of the service providers (Djelassi et al., 2018), along with the overall satisfaction derived from the experience (Chen et al., 2021). In light of the literature on word-of-mouth communication, it is evident that the perceived quality of products significantly impacts consumers’ word-of-mouth positivity (Ifie et al., 2018). Consumers are more inclined to express favorable thoughts about products of superior quality and providers that have met their satisfaction (Konuk, 2019). Given that exposure to cuteness increases consumer patience as a result of enhanced perceived social support in waiting scenarios, and patience is positively correlated with experience satisfaction, we propose that consumers exposed to cuteness are inclined to speak positively about products or service providers. Thus, we hypothesize:

*H4a*: Exposure to cuteness enhances word-of-mouth positivity.

*H4b*: Perceived social support and consumer patience serially mediate the effect of exposure to cuteness on word-of-mouth positivity.

## 3 Overview of studies

Five studies are conducted to examine our hypotheses, specifically focusing on how exposure to cuteness increases consumer patience through the mechanism of perceived social support. Study 1 demonstrates that consumers exhibit a tendency to wait longer for products with cute designs (H1). Study 2 replicates the main effect and reveals perceived social support as the underlying mechanism (H2) by employing cute visual stimuli and measuring consumer patience in the same domain. To bolster the underlying mechanism, Study 3 adopts a moderation approach by manipulating perceived social support (H3) and examines consumer patience in an unrelated domain. Specifically, it reveals that the cuteness effect is mitigated for consumers waiting with companions (vs. alone), for companionship denotes greater social support. Study 4 documents the moderating role that time pressure plays in the effect of cuteness on consumer patience (H3)^1^. Study 5 examines the subsequent consequence of consumer patience for word-of-mouth communication (H4a & H4b).

## 4 Study 1: the main effect of exposure to cuteness on consumer patience

### 4.1 Participants and design

The purpose of Study 1 was to offer preliminary evidence regarding the effect of exposure to cuteness on consumer patience using a one-factor (cute vs. control) between-subjects design. One hundred fifty participants (32% male, *M*_age_ = 28.93) were recruited from Credamo, a crowdsourcing platform (Chen et al., 2023; Li et al., 2023), in exchange for ¥1 RMB compensation (≈ $0.14 USD). Participants were assigned randomly to the cute condition (*N* = 75) or the control condition (*N* = 75).

### 4.2 Procedure

The first part of this study was an imagination task. Participants were first requested to imagine a promotional event in a new bakery located in their neighborhood. To attract customers, those who arrive at the bakery could get a box of cookies for free. Then, depending on the condition, they were shown a sample picture of either a cute cookie or a neutral cookie as the manipulation of cuteness (adapted from Nenkov and Scott (2014); see Supplementary Data Sheet 1). The cookies in both conditions look the same, except for the designs on the surface.

The second part was the measure of consumer patience. After viewing the picture, they were asked to imagine arriving at the bakery to pick up some free cookies but found a lengthy queue they had to wait in to get the free cookies. Participants utilized a slider with a range from 0 to 60 minutes to indicate the duration they were willing to wait (Thompson et al., 2020). The slider’s default position was set at zero, which suggested that they would not be willing to wait at all. The larger value they chose between 1 and 60, the more patient they were assumed to be. Finally, to check the effectiveness of the cuteness stimuli, participants rated to what extent they perceived the designs of the cookie as cute (1 = Not cute at all, 7 = Extremely cute).

### 4.3 Results and discussion

#### 4.3.1 Manipulation check

A one-way ANOVA demonstrated that participants exposed to the cute designs found the designs of the cookie to be cuter (*M_cute_* = 5.96, SD = 0.95) compared to those exposed to the neutral designs (*M_control_* = 4.68, SD = 1.69; *F* (1, 148) = 32.57, *p* < 0.001, *η*^2^ = 0.18), implying that the stimuli of cuteness were effective.

#### 4.3.2 Willingness to wait

Given the skewed distribution of the duration participants were willing to wait (skewness = 1.17), we conducted a one-way ANOVA with exposure to cuteness as the independent variable and the log-transformed willingness to wait as the dependent variable (Molden et al., 2012). In line with our hypothesis, a significant main effect of cuteness on consumer patience emerged (*F* (1, 148) = 5.8, *p* < 0.05, *η*^2^ = 0.04). Of note, the significance level did not change when using the untransformed data. For ease of understanding, the means derived from untransformed data were reported below. Specifically, participants exposed to the cute cookie (*M_cute_* = 23.55 min, SD = 13.83 min) showed a greater willingness to wait compared to those exposed to the neutral cookie (*M_control_* = 18.43 min, SD = 9.79 min; *F* (1, 148) = 5.8, *p* < 0.05, *η*^2^ = 0.04). Thus, H1 was supported.

#### 4.3.3 Discussion

By measuring consumers’ willingness to wait for products with cute (vs. neutral) designs, Study 1 provided initial support to our H1, such that exposure to cuteness increases consumer patience. However, one may argue that a similar effect may also present if one cookie shows a beautiful or interesting design, not necessarily cute. To clarify this possible confound, we manipulated cuteness using visual stimuli by levels of cuteness (high vs. low) that were irrelevant to the products in the next study. Additionally, we were about to explore the mediator underlying the cuteness effect.

## 5 Study 2: the mediating role of perceived social support

### 5.1 Pretest

Prior research has indicated that the cuteness effect can extend to animals (Kringelbach et al., 2016; Wang et al., 2017), which also serves as a significant source of social support (Beetz et al., 2012; Cacciatore et al., 2024). Therefore, we adopted pictures of animals (i.e., lions) to manipulate cuteness in Study 2. To verify the effectiveness of stimuli, a pretest was conducted among 107 participants (37.4% male, *M*_age_ = 29.3). Specifically, they were randomly shown four pictures of a lion cub (cute condition) or an adult lion (control condition) (see Supplementary Data Sheet 1). Participants were asked to rate the extent to which they found the animal cute, adorable, and endearing on a scale from 1 (Not at all) to 7 (Extremely) (Nenkov and Scott, 2014). The average of these three items was calculated to create an index of perceived cuteness (Cronbach’s α = 0.902). The results of the pretest showed that the pictures of the lion cub (*M_cute_* = 6.29, SD = 0.76) were considered cuter than those of the adult lion (*M_control_* = 4.5, SD = 1.53; *F* (1, 105) = 58.44, *p* < 0.001, *η*^2^ = 0.36), which means the stimuli were effective.

### 5.2 Participants and design

Study 2 aimed to replicate the main effect by separating the visual cuteness cues from the product or service that people were waiting for and to examine the mediating role of perceived social support. This study adopted a one-factor (cute vs. control) between-subjects design. One hundred and sixty participants (38.1% male, *M*_age_ = 28.4) were recruited from Credamo and received compensation of ¥1 RMB (≈ $0.14 USD). Participants were randomly assigned to either of the two conditions, with 80 in the cute condition and 80 in the control condition.

### 5.3 Procedure

To begin with, participants were instructed to read a scenario and immerse themselves in the perspective of the main character in that scenario. Specifically, they read the following excerpt, “A new restaurant in your neighborhood is having a huge promotional event. You arrive at the restaurant and find that there are so many people that you have to join a queue.” To help participants immerse in that scenario, they were shown a picture depicting the waiting scene. They were required to envision themselves as the last person in a line, which was indicated by a red arrow (see Supplementary Data Sheet 1). Subsequently, we manipulated cuteness by instructing them to imagine a documentary playing on a nearby TV while they were waiting in line. Participants were randomly shown the pictures of a lion cub (cute condition) or an adult lion (control condition) as verified in the pretest. To simulate the experience of watching a documentary, each picture remained on the screen for a minimum of 3 seconds before participants could advance to the next screen.

We then measured consumer patience by having participants indicate how patient they would feel while waiting in line (1 = Very impatient to 10 = Very patient; adapted from Huang et al., 2016). In the following step, they evaluated the extent to which they perceived social support from the animal in the documentary on the following three items (adapted from Zimet et al., 1990; Cronbach’s *α* = 0.918): “I think I can: (a) share my joys and sorrows with it, (b) get emotional help and support from it, and (c) get comfort from it” (1 = Strongly disagree to 7 = Strongly agree). Finally, participants responded to a manipulation check with the same three items as in the pretest (Nenkov and Scott, 2014; Cronbach’s *α* = 0.930).

### 5.4 Results and discussion

#### 5.4.1 Manipulation check

Results of a one-way ANOVA confirmed that participants who were shown the pictures of a lion cub perceived the pictures as cuter (*M_cute_* = 6.21, SD = 0.63) than those exposed to the pictures of an adult lion (*M_control_* = 4.15, *SD* = 1.43; *F* (1, 158) = 139.77, *p* < 0.001, *η*^2^ = 0.04), indicating the successful manipulation of cuteness.

#### 5.4.2 Consumer patience

As we predicted, the findings demonstrated that in comparison to participants exposed to images of the adult lion in the control condition (*M_control_* = 5.64, SD = 1.96), those exposed to the lion cub in the cute condition reported being significantly more patient (*M_cute_* = 6.44, SD = 2.13; *F* (1, 158) = 6.11, *p* < 0.05, *η*^2^ = 0.04) while waiting in the given scenario. Therefore, H1 was again supported.

#### 5.4.3 The mediating role of perceived social support

**Figure 1:**
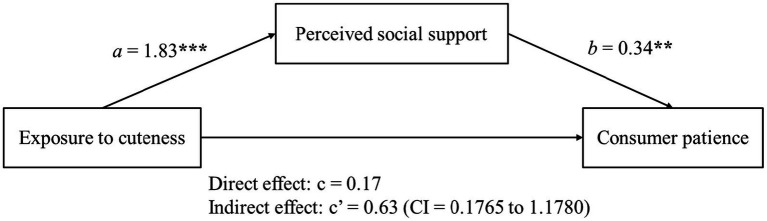
The mediating role of perceived social support in Study 2.

To verify whether perceived social support mediated the effect of exposure to cuteness on patience, we ran a mediational analysis using the PROCESS (Model 4; Hayes, 2017) with cuteness as the independent variable, perceived social support as the mediator, and consumer patience as the dependent variable. The results revealed that cuteness (control = −1, cute = 1) had a positive correlation with perceived social support (*β* = 1.83, *t* = 8.11, *p* < 0.001), while perceived social support also has a positive association with consumer patience (*β* = 0.34, *t* = 3.1, *p* < 0.01). Controlling for perceived social support, the effect of cuteness on consumer patience was found to be non-significant (*β* = 0.17, *t* = 0.45, *p* > 0.1). Moreover, mediation analysis confirmed that the indirect effect of perceived social support on consumer patience was significant (5000 samples, 95% CI = [0.1765 to 1.1780], excluding 0; see Figure 1). Thus, H2 was supported.

#### 5.4.4 Discussion

The findings from Study 2 offered compelling evidence for the main effect, employing visual cuteness cues that were separate from the product or service that people waited for, though they were still in the same domain. Additionally, perceived social support was identified as the mediator in the effect of cuteness on consumer patience. Perceived social support elicited by exposure to cuteness, in turn, led to great consumer patience. The next study would further show the robustness of this underlying process by manipulating perceived social support and exploring the effect of cuteness on consumer patience present in an unrelated domain.

## 6 Study 3: bolstering the mediating effect of perceived social support with a moderation approach

### 6.1 Participants and design

In order to bolster the underlying mechanism, Study 3 utilized a moderation approach by manipulating perceived social support. Prior research has demonstrated that individuals perceive greater social support when shopping with companions as opposed to shopping alone (Lucia-Palacios et al., 2018). Thus, we hypothesized that the effect of cuteness on consumer patience would be mitigated when people wait with a companion (high perceived social support), while the effect remains when they wait alone (low perceived social support). Also, this study aimed to examine how incidental exposure to cuteness would influence consumer patience in an unrelated domain. We used a 2 (cuteness: cute vs. control) × 2 (perceived social support: companion vs. alone) between-subjects design in this study. Two hundred eighty participants (36.1% male, *M*_age_ = 29.56) were recruited from Credamo for remuneration of ¥1 RMB (≈ $0.14 USD). They were randomly assigned to one of these four conditions: cute/high perceived social support condition (*N* = 70), cute/low perceived social support condition (*N* = 71), control/high perceived social support condition (*N* = 70), and control/low perceived social support condition (*N* = 69).

### 6.2 Procedure

To begin with, participants were informed to perform a picture memory task, which served as the manipulation of cuteness. They were told they would view four pictures of an animal and were required to remember its features as much as possible to perform better in the later memory test. As in Study 2, they were shown four pictures of a lion cub (cute condition) or an adult lion (control condition) depending on the condition. Then they were required to select the pictures that they had just seen among five pictures (an additional picture was included to justify the cover story) and to evaluate the pictures by responding to three items measuring perceived cuteness as in Study 2 (Cronbach’s α = 0.937).

Next, we manipulated perceived social support by asking participants to imagine themselves waiting with a companion (high perceived social support condition) or alone (low perceived social support condition). We asked participants in the companion condition to write initials of a friend or a family member with whom they were very close. Then, they were given a waiting scenario that was different from those in the previous studies: “Imagine you are waiting at a bus stop with XX (the initials of their friend or family).” Meanwhile, participants in the alone condition were not instructed to recall significant others. Instead, they were solely asked to imagine the scenario where they were waiting at the bus stop alone. Participants were shown a picture depicting the bus stop (see Supplementary Data Sheet 1) in both conditions to help them better imagine the scenario. We next asked all the participants to rate how patient they would feel when they waited for the bus (1 = Very impatient to 10 = Very patient; Huang et al., 2016).

To make sure the manipulation of perceived social support was successful, we conducted an independent pretest using 90 participants from the same population as those in the main study. The results suggested that compared with waiting alone (*M_alone_* = 4.35, SD = 1.77), participants who imagined waiting for a bus with a friend or a family member perceived greater social support (*M*_companion_ = 5.93, SD = 0.81; *F* (1, 88) = 29.7, *p* < 0.001, *η*^2^ = 0.25), confirming the successful manipulation of perceived social support.

### 6.3 Results and discussion

#### 6.3.1 Manipulation check

A one-way ANOVA revealed that participants perceived the pictures of a lion cub as cuter (*M_cute_* = 6.29, SD = 0.73) than those of an adult lion (*M_control_* = 3.96, SD = 1.44; *F* (1, 278) = 291.93, *p* < 0.001, *η*^2^ = 0.51), confirming that the manipulation worked as intended.

#### 6.3.2 Consumer patience

Results of a 2 (cuteness) × 2 (perceived social support) ANOVA revealed a marginally significant interaction between cuteness and perceived social support (*F* (1, 276) = 3.34, *p* = 0.069, *η*^2^ = 0.01) and a significant main effect of perceived social support (*F* (1, 276) = 80, *p* < 0.001, *η*^2^ = 0.2). Results of further planned contrasts showed, for those in the alone (low social support) condition, consumer patience was greater in the cute condition compared to the control condition (*M*_cute_ = 6.42, SD = 2.05; *M*_control_ = 5.72, SD = 2.13; *F* (1, 276) = 4.76, *p* < 0.05, *η*^2^ = 0.02). Conversely, for those in the companion (high social support) condition, no significant difference in consumer patience was found between the cute and control conditions (*M*_cute_ = 7.91, SD = 1.68; *M*_control_ = 8.04, SD = 1.65; *F* (1, 276) = 0.16, *p* > 0.1).

#### 6.3.3 Discussion

By employing a moderation approach, the findings of Study 3 indicated a condition that increased people’s perceived social support mitigated the impact of cuteness on consumer patience. The findings have bolstered the proposed mechanism of perceived social support underlying the main prediction. Moreover, using a cuteness-unrelated waiting domain attested to the robustness of our proposed effect and further supported that incidental exposure to cuteness can also increase consumer patience. In addition, previous research found that exposure to cuteness is positively associated with positive mood (Shin and Mattila, 2021), which might also lead to greater patience (Pyone and Isen, 2011). In Study 4, we were about to eliminate the alternative explanation of mood while testing the moderator, time pressure.

## 7 Study 4: the moderating role of time pressure

### 7.1 Participants and design

The purpose of Study 4 was to examine the robustness of our theory by testing time pressure as a moderator. Specifically, we predicted that the influence of exposure to cuteness on consumer patience would be evident only among participants waiting under low time pressure and disappears for those under high time pressure (H3). Meanwhile, we focused on a new context, i.e., in-store shopping, to replicate the finding, providing additional managerial implications. Moreover, we ruled out the possible mediating effect of mood.

Study 4 was a preregistered study (see text footnote 1) with a planned target sample size of 300 participants. A 2 (cuteness: cute vs. control) × 2 (time pressure: high vs. low) between-subjects design was employed. Three hundred and one participants were recruited from Prolific for reimbursement of approximately $0.40 USD. After the exclusion of 11 participants who did not pass the attention check, we analyzed data from 290 participants (49.7% male, *M*_age_ = 39.23) for subsequent analyses. Of note, the results did not change significantly after the exclusion. Participants were randomly assigned to one of these four conditions: cute/high time pressure condition (*N* = 70), cute/low time pressure condition (*N* = 73), control/high time pressure condition (*N* = 76), and control/low time pressure condition (*N* = 71).

### 7.2 Procedure

All participants were firstly instructed to imagine a new retail store in their neighborhood, and they went to that store but found a lot of people lining up to enter the store. They were shown a real picture of people waiting in line to better imagine the scenario (see Supplementary Data Sheet 1). To manipulate cuteness, they imagined that they stood in the line and noticed some animal posters displayed on the store window. They were randomly shown four pictures of a lion as in Studies 2 and 3. Regarding time pressure, participants in the high time pressure condition were required to imagine that they went to that store on a busy day. As they waited in line, they realized they needed to hurry to an important appointment afterward. On the contrary, those in the low time pressure condition imagined they went to that store on a normal day and they were not reminded of anything coming up. Consumer patience was then measured by asking them to rate both how patient they would feel as they waited to enter the store (1 = Very impatient, 10 = Very patient) and the extent to which they would wait patiently to enter the store (1 = Not at all, 10 = Very much). For analyses, we averaged the responses of these two items as an index of consumer patience (adapted from Huang et al., 2016; *r* = 0.865).

The second part was a design evaluation task serving as the manipulation check of cuteness. Participants were shown the pictures of the lion cub and the adult lion and rated their perceived cuteness with three items as in the prior studies. To verify the effectiveness of the manipulation of time pressure, they indicated how much time pressure they felt while waiting in line (1 = Not at all, 7 = Very much; Dhar and Nowlis, 1999). In addition, four items were used to measure their mood (Song et al., 2017), involving positive mood (i.e., happy and joyful; *r* = 0.899) and negative mood (i.e., angry and sad; *r* = 0.658). Next, participants were instructed to answer an attention check question by ignoring a simple math problem (“9–3 =?”) and not check any answer. Those who selected any answer were not included in the analysis. Finally, they indicated their liking of animals (1 = Not at all, 7 = Very much), which was used as the control variable for subsequent analyses.

### 7.3 Results and discussion

#### 7.3.1 Manipulation check

The results of a one-way ANOVA revealed that participants perceived the pictures of a lion cub as cuter (*M*_cute_ = 6.42, SD = 0.81) than those of an adult lion (*M*_control_ = 4.44, SD = 1.51; *F* (1, 288) = 192.50, *p* < 0.001, *η*^2^ = 0.40), confirming that the stimuli of cuteness were effective. Also, participants reported that they perceived greater time pressure in the high time pressure condition (*M*_high_ = 5.68, SD = 1.66) in contrast to those in the low time pressure condition (*M*_low_ = 4.21, *SD* = 1.75; *F* (1, 288) = 54.50, *p* < 0.001, *η*^2^ = 0.16). This indicated that the manipulation of time pressure was successful.

#### 7.3.2 Consumer patience

Results of a 2 (cuteness) × 2 (time pressure) ANOVA revealed a significant interaction between cuteness and time pressure (*F* (1, 286) = 5.90, *p* < 0.05, *η*^2^ = 0.02) and a significant main effect of time pressure (*F* (1, 286) = 100.63, *p* < 0.001, *η*^2^ = 0.26). We conducted planned contrasts in order to investigate the significant interaction. Specifically, for those in the low time pressure condition, participants exposed to cute images reported a heightened patience in contrast to those in the control condition (*M*_cute_ = 5.88, SD = 2.41; *M*_control_ = 5.08, SD = 2.47; *F* (1, 286) = 4.51, *p* < 0.05, *η*^2^ = 0.02). Conversely, those facing high time pressure showed no significant difference in consumer patience between the cute and control conditions (*M*_cute_ = 2.60, SD = 1.93; *M*_control_ = 3.09, SD = 2.10; *F* (1, 286) = 1.72, *p* > 0.1). Of note, the significant interaction between cuteness and time pressure persisted even after accounting for the liking of animals (*F* (1, 285) = 6.01, *p* < 0.05, *η*^2^ = 0.02). H3 was supported.

#### 7.3.3 Mood

A 2 (cuteness) × 2 (time pressure) ANOVA showed that the main effect of cuteness on positive and negative moods was not significant, nor was the interaction between cuteness and time pressure (*p*s > 0.1). Therefore, the alternative explanation of mood was ruled out.

#### 7.3.4 Discussion

From the results of Study 4, it was demonstrated that the effect of cuteness on consumer patience remained in situations where consumers experience low time pressure. Conversely, for consumers shopping with high time pressure, the effect was diminished. Furthermore, we ruled out mood as a potential alternative explanation and controlled for participants’ liking of animals, enhancing the robustness of our proposed mechanism. To provide more marketing implications, we would identify the downstream consequence of consumer patience for word-of-mouth positivity in the next study.

## 8 Study 5: exposure to cuteness influences word-of-mouth positivity via perceived social support and consumer patience

### 8.1 Pretest

In Study 5, we manipulated cuteness using a queue ticket featuring an image of an elephant, with variations in cuteness levels (high vs. low; see Supplementary Data Sheet 1). To confirm the effectiveness of this manipulation, the images were pretested with 90 participants (50% male, *M*_age_ = 36.04). Participants were instructed to evaluate either a cartoon (cute condition) or a realistic drawing (control condition) of an elephant, rating the perceived cuteness on three items as in prior studies. Also, they indicated the innocence and attractiveness of the animal. Results of a one-way ANOVA confirmed that participants perceived the cartoon as cuter than the realistic drawing (*M*_cute_ = 5.39, *SD* = 1.21; *M*_control_ = 4.37, SD = 1.54; *F* (1, 88) = 12.26, *p* = 0.001, *η*^2^ = 0.12). Meanwhile, there was no significant difference in innocence (*M*_cute_ = 5.29, SD = 1.25; *M*_control_ = 5.09, *SD* = 1.43; *F* (1, 88) = 0.50, *p* > 0.1) or attractiveness (*M*_cute_ = 4.22, SD = 1.68; *M*_control_ = 4.24, SD = 1.60; *F* (1, 88) = 0.004, *p* > 0.1) of these two animals.

### 8.2 Participants and design

Study 5 was designed to explore how exposure to cuteness influences word-of-mouth positivity, with perceived social support and consumer patience acting as the underlying mechanism (H4a & H4b). One hundred and fifty participants (36% male, *M*_age_ = 31.68) were recruited from Credamo for remuneration of approximately $0.14 USD. We employed a one-factor (cute vs. control) between-subjects design and randomly assigned the participants into either the cute condition (*N* = 75) or the control condition (*N* = 75).

### 8.3 Procedure

Participants were asked to imagine a waiting scenario similar to Study 2. To manipulate cuteness, they were told to imagine that as they waited in line, a server handed them a queue ticket adorned with either an elephant cub (cute condition) or an adult elephant (control condition) as in the pretest. To measure consumer patience, participants were requested to answer two questions similar to those in Study 4: “How patient would you feel as you wait to be seated? (1 = Very impatient, 10 = Very patient),” and “To what extent would you wait patiently for the table? (1 = Not at all, 10 = Very much).” We then averaged the responses to form an index of consumer patience (adapted from Huang et al., 2016; 2 items, *r* = 0.897).

Regarding word-of-mouth positivity, participants responded to the following statements: (1) I have good things to say about this restaurant, (2) I will recommend that others eat in this restaurant (1 = Strongly disagree, 7 = Strongly agree). They also rated the likelihood of telling friends and acquaintances positive things about this restaurant (1 = Not likely at all, 7 = Very likely). To create an index of word-of-mouth positivity, we averaged the responses to these three items (adapted from Cheema and Kaikati, 2010; Cronbach’s *α* = 0.898). Next, participants were instructed to the manipulation check of cuteness on the queue ticket as in the pretest (3 items, Cronbach’s *α* = 0.930). They also rated the extent to which they perceived social support from the animal on three items as in Study 2 (Cronbach’s *α* = 0.925). Finally, they responded to demographic questions.

### 8.4 Results and discussion

#### 8.4.1 Manipulation check

The results of a one-way ANOVA revealed that participants exposed to the cute queue ticket perceived the animal as cuter (*M*_cute_ = 6.07, SD = 0.78) than those exposed to the queue ticket in the control condition (*M_control_* = 4.60, SD = 1.45; *F* (1, 148) = 60.38, *p* < 0.001, *η*^2^ = 0.29), implying that the manipulation of cuteness was successful.

#### 8.4.2 Word-of-mouth positivity

A significant main effect of exposure to cuteness on word-of-mouth positivity was indicated by the results, such that participants exposed to the cute queue ticket had a greater intention to share positive thoughts about the restaurant than those exposed to the queue ticket in the control condition (*M_cute_* = 4.72, SD = 1.29; *M_control_* = 4.16, SD = 1.43; *F* (1, 148) = 6.18, *p* < 0.05, *η*^2^ = 0.04). Thus, H4a was supported.

#### 8.4.3 Consumer patience

We conducted a one-way ANOVA with cuteness as the independent variable and consumer patience as the dependent variable. Consistent with the hypothesis, a significant effect of cuteness emerged (*F* (1, 148) = 5.18, *p* < 0.05, *η*^2^ = 0.03). Specifically, participants exposed to the cute queue ticket in the cute condition (*M_cute_* = 5.40, SD = 2.27) reported being more patient than those exposed to the ticket in the control condition (*M_control_* = 4.55, *SD* = 2.28), again confirming H1.

#### 8.4.4 Serial mediation effect of perceived social support and consumer patience

**Figure 2:**
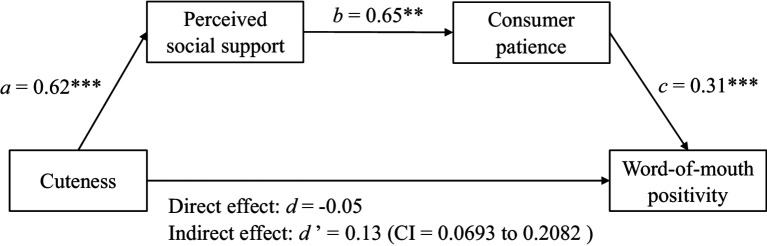
The serial mediation effect of perceived social support and consumer patience.

To test the mediating roles of perceived social support and consumer patience, we conducted a serial mediation analysis using Process with cuteness (control = −1, cuteness = 1) as the independent variable, perceived social support and consumer patience as the serial mediators, and word-of-mouth positivity as the dependent variable (Model 6; Hayes, 2017). As shown in Figure 2, cuteness increased perceived social support (*β* = 0.62, *t* = 5.28, *p* < 0.001), perceived social support enhanced consumer patience (*β* = 0.65, *t* = 5.44, *p* < 0.001), and consumer patience positively influenced word-of-mouth positivity (*β* = 0.31, *t* = 8.30, *p* < 0.001). Moreover, we found a significant indirect effect through perceived social support and consumer patience as serial mediators (5000 samples, 95% CI = [0.0693 to 0.2082], excluding 0). Therefore, H4b was supported.

#### 8.4.5 Discussion

Focusing on a more consequential outcome variable, i.e., word-of-mouth positivity, Study 5 provided important practical implications of cuteness for marketing. The results demonstrated that consumers exposed to cuteness were inclined to share favorable opinions about the product or experience they waited for, as a result of their elevated perceived social support and patience. The robustness of the mechanism we proposed was further strengthened.

## 9 General discussion

The current research proposes a causal relationship between exposure to cuteness and consumer patience in waiting scenarios based on theories of priming, attachment theory, and conservation of resources theory. Across five experiments, we find that exposure to cuteness increases consumer patience in subsequent waiting scenarios, including waiting in line to pick up a free gift in a bakery (Study 1), waiting to be seated in a restaurant (Studies 2 and 5), waiting for a bus (Study 3), and waiting to enter a retail store (Study 4). Furthermore, we demonstrate that the effect of cuteness is driven by perceived social support employing both mediation (Study 2) and moderation approaches (Study 3). In addition, we investigate the moderating role of time pressure in the cuteness effect (Study 4). Specifically, the effect of cuteness on consumer patience only exists in participants under low time pressure and disappears for those under high time pressure. Given that exposure to cuteness increases perceived social support and subsequent consumer patience, we further identify word-of-mouth positivity as the downstream consequence (Study 5). Our work employs a variety of stimuli to manipulate cuteness, including cute product designs (Study 1), pictures of baby animals (Studies 2, 3, and 4), and cute queue tickets (Study 5). Robustness and validity are hence enhanced.

### 9.1 Theoretical contributions

The findings of our research make significant contributions to the existing literature in several aspects. Firstly, our research sheds new light on the cuteness literature, demonstrating the positive effect of cuteness on consumer patience, which is generally considered high levels of self-control (Khan and Dhar, 2007). Our findings deepen the understanding of cuteness by highlighting its impact on behaviors related to self-control, such as consumer patience. Additionally, while previous studies emphasize the care-releasing function of cuteness, such as bringing about more sustainable behavior (Wang et al., 2017) and prosocial behavior (Shin and Mattila, 2021), the current research focuses on the sociality-releasing function of cuteness, as suggested by Sherman and Haidt (2011). The findings of the current research are a valuable addition to the literature, wherein cuteness can be treated as a trigger of sociality, which, in turn, has brings about a positive impact on its downstream consequences of consumer patience and word-of-mouth positivity.

Secondly, by introducing exposure to cuteness as a new situational factor, we demonstrate its positive impact on consumer patience exhibited in time-consuming waiting scenarios. While previous literature on consumer patience mostly centers on patience in intertemporal choices concerning trade-offs between time and benefit (i.e., smaller-sooner vs. larger-later; May and Monga, 2014; Huang et al., 2016; Shaddy and Lee, 2020), there is little understanding of consumers’ reactions to frequently encountered and mentally taxing waiting scenarios, where consumers spend a considerable amount of time with no benefit increased. Regarding this gap, our research looks into various waiting scenarios, such as waiting for a free gift, waiting to be seated in a restaurant, waiting for a bus, and waiting to enter a retail store. Besides, although much research has examined situational factors that lead to impatience (Kim and Zauberman, 2019; Shaddy and Lee, 2020), to our best knowledge, few situational factors have been found to increase consumer patience. Consequently, the present research enriches the consumer patience literature by focusing on ubiquitous yet overlooked waiting scenarios and proposing exposure to cuteness as a novel situational antecedent that enhances consumer patience.

Thirdly, our work broadens the sources of social support and reveals its positive effect on consumer patience, meanwhile advancing the application of the attachment theory and conservation of resources theory in the marketing domain. Although prior research has noted family and friends as primary sources of social support (Gurrieri and Drenten, 2019), sometimes people might perceive them as judgmental and thus experience a stressor (Allen, 2003). By contrast, given the non-threatening and supportive nature of cute agents (Nicholas and Gullone, 2001), attachment theory and the developmental psychology literature suggest that individuals may perceive social support from them since childhood, as they serve as substitutes for their primary attachment figures during times of separation from their caregivers (Nicholas and Gullone, 2001; Bowlby and Holmes, 2012). Additionally, prior research has found that perceived social support reduces both physical pain and spending pain (Zhou and Gao, 2008; Xu et al., 2015). Our findings uncover that it may also be able to counteract the pain of waiting, supporting the resource substitution hypothesis, which suggests different resources (e.g., social support, time, money, and energy) can be substituted for one another in addressing the challenges posed by the (potential) loss of the latter (Hobfoll et al., 1990).

### 9.2 Managerial implications

The current research yields three noteworthy implications for marketers. First, as waiting becomes increasingly prevalent in marketing scenarios, our findings indicate that consumer patience significantly enhances consumer word-of-mouth positivity. Marketers should recognize that perceived social support elicited by exposure to cuteness can increase consumer patience during waiting times or service delays. Further, when consumers are more willing to wait patiently for the chosen products or services, they are more likely to speak positively about them with friends, family, and online communities. According to our findings, marketers can promote positive word-of-mouth by incorporating cuteness cues in waiting scenarios to foster consumer patience.

Second, the current research offers valuable insights to guide marketers in making decisions regarding the design of products, waiting areas, and service supplements to increase consumer patience. Specifically, for products that consumers have to wait for, marketers could add cute elements to the design of such products to increase consumers’ willingness to wait patiently for the product to be served. Meanwhile, in situations where consumers need to wait for services, such as waiting to be seated in a restaurant and waiting to enter a retail store, marketers can enhance consumer patience by incorporating cute elements in various communication touchpoints, involving displaying cute posters on store windows, showing videos or vignettes featuring baby animals in the waiting lounges, and providing consumers with cute queue tickets when they are lining up. While previously marketers have to offer monetary incentives to encourage consumers to wait to be served, our findings provide a cost-efficient means of using visual cuteness cues to enhance consumer patience, which is practical, easy to handle, and cost-saving.

Third, drawing on our findings, marketers can develop more effective and targeted marketing strategies by considering the moderation effect of time pressure. Since our results suggest that exposure to cuteness increases consumer patience only for those under low time pressure, marketers could take consumers’ time constraints into account when leveraging the power of cuteness. For example, marketers should be aware that the effectiveness of cuteness may be mitigated for time-pressed consumers or during the shopping festival rush (i.e., Black Friday).

### 9.3 Limitations and future research

This research also has several limitations, indicating suggestions for future research. Firstly, regarding the concept of cuteness, there are two types of cuteness suggested by Nenkov and Scott (2014), which are kindchenschema cuteness and whimsical cuteness. While the latter is linked to unpredictable humor and a playful temperament (e.g., a dots gift card), the current research focuses on the kindchenschema cuteness, given its theoretical clarity, which is conducive to ensuring the internal validity and providing specific implications for marketing practice. Future investigation is called for to examine if the effect exists for whimsical cuteness. Additionally, concerning the manipulation of cuteness, although we have employed diverse manipulations to ensure the robustness and validity of our findings, all the manipulations used the same method, i.e., viewing cute images. According to Shin and Mattila (2021), listening to cute sounds can also be an effective manipulation approach. Thus, future research could adopt cute sounds as stimuli to determine whether our findings still hold.

Secondly, this research has investigated the positive association between exposure to cuteness and consumer patience in different waiting scenarios, including waiting in line to pick up a gift, waiting to be seated in a restaurant, waiting for a bus, and waiting to enter a retail store. Nevertheless, there are many other waiting scenarios that need to be tested (Djelassi et al., 2018; Witowska et al., 2020). To go further, future research could examine whether the effect is present in other common scenarios, such as waiting for checkout and online waiting for assistance. Additionally, existing literature shows that consumer patience is defined not only as the willingness to wait but also as patience in making an intertemporal choice (Bartels and Urminsky, 2011; Huang et al., 2016; Romero et al., 2019). Hence, future studies may explore whether the proposed framework and conceptualization explain consumers’ being patient in choosing a later option to gain larger benefits instead of a smaller-sooner one after exposure to cuteness.

Thirdly, while this study contributes valuable insights into the concept of cuteness and its effects, it is essential to acknowledge several methodological limitations inherent in the experimental design. One of the primary limitations of this study pertains to history errors. Despite efforts to control extraneous variables and maintain consistency throughout the experimental procedures, external factors such as current events or personal experiences could have impacted participants’ responses to cuteness, thereby confounding the results. Future research could control for history errors by carefully recording relevant information about participants before the experiment. Additionally, caution may be warranted in interpreting our findings due to the scenario-based measures of consumer patience, which might diverge from actual consumer behavior. Future research may enhance external validity by incorporating field experiments and observations of real behaviors.

Finally, though we have recognized time pressure as a moderator for the effect of exposure to cuteness on consumer patience, it is critical to identify more boundary conditions for the focal effect. For example, the cuteness effect may be less pronounced for consumers with a utilitarian (vs. hedonic) shopping motivation. Similarly, cuteness cues may not fit for all product categories, i.e., financial products, medical services. These moderators warrant future investigation.

## Data availability statement

The raw data supporting the conclusions of this article will be made available by the authors, without undue reservation.

## Ethics statement

The studies involving humans were approved by School of Business, Renmin University of China. The studies were conducted in accordance with the local legislation and institutional requirements. The participants provided their written informed consent to participate in this study.

## Author contributions

XW: Conceptualization, Data curation, Formal analysis, Writing – original draft, Writing – review & editing. JJ: Conceptualization, Supervision, Writing – original draft, Writing – review & editing. XZ: Conceptualization, Data curation, Formal analysis, Writing – original draft, Writing – review & editing.

## Footnotes

^1^ Study 4 was a preregistered study. The preregistered report can be found at https://aspredicted.org/hg842.pdf

## References

[ref1] Allen K. (2003). Are pets a healthy pleasure? The influence of pets on blood pressure. Curr. Dir. Psychol. Sci. 12, 236–239. doi: 10.1046/j.0963-7214.2003.01269.x

[ref2] Bargh J. A. Bond R. N. Lombardi W. J. Tota M. E. (1986). The additive nature of chronic and temporary sources of construct accessibility. J. Pers. Soc. Psychol. 50, 869–878. doi: 10.1037/0022-3514.50.5.869

[ref3] Bartels D. M. Urminsky O. (2011). On intertemporal selfishness: how the perceived instability of identity underlies impatient consumption. J. Consum. Res. 38, 182–198. doi: 10.1086/658339

[ref4] Baumeister R. F. Leary M. R. (2017). “The need to belong: desire for interpersonal attachments as a fundamental human motivation” in Interpersonal development. ed. Zukauskiene R. (London: Routledge), 57–89. 7777651

[ref9001] Beetz A. Julius H. Turner D. Kotrschal K. (2012). Effects of social support by a dog on stress modulation in male children with insecure attachment. Front. Psychol. 3:352. doi: 10.3389/fpsyg.2012.00352 23162482 PMC3498889

[ref5] Belch G. E. Belch M. A. (2004). Advertising and promotion: an integrated marketing communications perspective. New York: McGraw-Hill.

[ref6] Bowlby J. Holmes J. (2012). A secure base. New York: Routledge.

[ref7] Brosch T. Sander D. Scherer K. R. (2007). That baby caught my eye… attention capture by infant faces. Emotion 7, 685–689. doi: 10.1037/1528-3542.7.3.685, PMID: 17683225

[ref9002] Cacciatore J. Gorman R. Thieleman K. Sullivan M. (2024). “I don’t feel judged. i just feel love”: perceptions of animals as support for grievers Soc. Animals. 1–19. doi: 10.1163/15685306-bja10178

[ref8] Cheema A. Kaikati A. M. (2010). The effect of need for uniqueness on word of mouth. J. Mark. Res. 47, 553–563. doi: 10.1509/jmkr.47.3.553

[ref9] Chen Q. Wang Y. Ordabayeva N. (2023). The mate screening motive: how women use luxury consumption to signal to men. J. Consum. Res. 50, 303–321. doi: 10.1093/jcr/ucac034

[ref10] Chen S. Wei H. Ran Y. Li Q. Meng L. (2021). Waiting for a download: the effect of congruency between anthropomorphic cues and shopping motivation on consumer patience. Psychol. Mark. 38, 2327–2338. doi: 10.1002/mar.21564

[ref9003] Curry O. S. Price M. E. Price J. G. (2008). Patience is a virtue: cooperative people have lower discount rates. Pers. Individ. Dif. 44, 780–785. doi: 10.1016/j.paid.2007.09.023

[ref11] Dai X. Fishbach A. (2013). When waiting to choose increases patience. Organ. Behav. Hum. Decis. Process. 121, 256–266. doi: 10.1016/j.obhdp.2013.01.007

[ref12] Dhar R. Nowlis S. M. (1999). The effect of time pressure on consumer choice deferral. J. Consum. Res. 25, 369–384. doi: 10.1086/209545

[ref13] Dijksterhuis A. Bargh J. A. (2001). The perception-behavior expressway: automatic effects of social perception on social behavior. Adv. Exp. Soc. Psychol. 33, 1–40. doi: 10.1016/S0065-2601(01)80003-4

[ref14] Djelassi S. Diallo M. F. Zielke S. (2018). How self-service technology experience evaluation affects waiting time and customer satisfaction? A moderated mediation model. Decis. Support. Syst. 111, 38–47. doi: 10.1016/j.dss.2018.04.004

[ref15] Fiske A. P. Seibt B. Schubert T. (2019). The sudden devotion emotion: kama muta and the cultural practices whose function is to evoke it. Emot. Rev. 11, 74–86. doi: 10.1177/1754073917723167

[ref16] Fortuna K. Baor L. Israel S. Abadi A. Knafo A. (2014). Attachment to inanimate objects and early childcare: a twin study. Front. Psychol. 5:486. doi: 10.3389/fpsyg.2014.00486, PMID: 24904499 PMC4033092

[ref17] Garaus M. Wagner U. (2019). Let me entertain you–increasing overall store satisfaction through digital signage in retail waiting areas. J. Retail. Consum. Serv. 47, 331–338. doi: 10.1016/j.jretconser.2018.12.008

[ref18] Gjersoe N. L. Hall E. L. Hood B. (2015). Children attribute mental lives to toys when they are emotionally attached to them. Cogn. Dev. 34, 28–38. doi: 10.1016/j.cogdev.2014.12.002

[ref19] Glocker M. L. Langleben D. D. Ruparel K. Loughead J. W. Gur R. C. Sachser N. (2009). Baby schema in infant faces induces cuteness perception and motivation for caretaking in adults. Ethology 115, 257–263. doi: 10.1111/j.1439-0310.2008.01603.x, PMID: 22267884 PMC3260535

[ref20] Gorn G. J. Jiang Y. Johar G. V. (2008). Babyfaces, trait inferences, and company evaluations in a public relations crisis. J. Consum. Res. 35, 36–49. doi: 10.1086/529533

[ref21] Gui D.-Y. Liu S. Dai Y. Liu Y. Wang X. Huang H. (2021). Greater patience and monetary expenditure: how shopping with companions influences purchase decisions. J. Retail. Consum. Serv. 63:102665. doi: 10.1016/j.jretconser.2021.102665

[ref22] Gurrieri L. Drenten J. (2019). Visual storytelling and vulnerable health care consumers: normalising practices and social support through Instagram. J. Serv. Mark. 33, 702–720. doi: 10.1108/JSM-09-2018-0262

[ref23] Hayes A. F. (2017). Introduction to mediation, moderation, and conditional process analysis: A regression-based approach. New York: Guilford Press.

[ref24] Hobfoll S. E. (1989). Conservation of resources: a new attempt at conceptualizing stress. Am. Psychol. 44, 513–524. doi: 10.1037/0003-066X.44.3.513, PMID: 2648906

[ref25] Hobfoll S. E. Freedy J. Lane C. Geller P. (1990). Conservation of social resources: social support resource theory. J. Soc. Pers. Relat. 7, 465–478. doi: 10.1177/0265407590074004

[ref26] Hobfoll S. E. Stokes J. P. (1988). “The process and mechanics of social support” in Handbook of personal relationships: Theory, research and interventions. eds. Duck S. Hay D. F. Hobfoll S. E. Ickes W. Montgomery B. M. (Hoboken, NJ: John Wiley & Sons), 497–517.

[ref27] Huang X. Huang Z. Wyer R. S. Jr. (2016). Slowing down in the good old days: the effect of nostalgia on consumer patience. J. Consum. Res. 43, 372–387. doi: 10.1093/jcr/ucw033

[ref28] Huddleston P. T. Behe B. K. Driesener C. Minahan S. (2018). Inside-outside: using eye-tracking to investigate search-choice processes in the retail environment. J. Retail. Consum. Serv. 43, 85–93. doi: 10.1016/j.jretconser.2018.03.006

[ref29] Hughes A. (2023). Internet use has left most Britons with no patience for waiting in line, survey says. Available at: https://www.independent.co.uk/news/uk/home-news/internet-britons-patience-waiting-a8884686.html.

[ref30] Ifie K. Simintiras A. C. Dwivedi Y. Mavridou V. (2018). How service quality and outcome confidence drive pre-outcome word-of-mouth. J. Retail. Consum. Serv. 44, 214–221. doi: 10.1016/j.jretconser.2018.07.002

[ref31] Iyer E. S. (1989). Unplanned purchasing: knowledge of shopping environment and time pressure. J. Retail. 65, 40–57.

[ref32] Khan U. Dhar R. (2007). Where there is a way, is there a will? The effect of future choices on self-control. J. Exp. Psychol. Gen. 136, 277–288. doi: 10.1037/0096-3445.136.2.277, PMID: 17500651

[ref33] Kim B. K. Zauberman G. (2013). Can Victoria’s secret change the future? A subjective time perception account of sexual-cue effects on impatience. J. Exp. Psychol. Gen. 142, 328–335. doi: 10.1037/a0028954, PMID: 22686639

[ref34] Kim K. Zauberman G. (2019). The effect of music tempo on consumer impatience in intertemporal decisions. Eur. J. Mark. 53, 504–523. doi: 10.1108/EJM-10-2017-0696

[ref35] Konuk F. A. (2019). The influence of perceived food quality, price fairness, perceived value and satisfaction on customers’ revisit and word-of-mouth intentions towards organic food restaurants. J. Retail. Consum. Serv. 50, 103–110. doi: 10.1016/j.jretconser.2019.05.005

[ref36] Kringelbach M. L. Stark E. A. Alexander C. Bornstein M. H. Stein A. (2016). On cuteness: unlocking the parental brain and beyond. Trends Cogn. Sci. 20, 545–558. doi: 10.1016/j.tics.2016.05.003, PMID: 27211583 PMC4956347

[ref37] Lee J. Yoon S.-Y. (2023). Nature and patient waiting: mediating effects of anxiety and perceived wait time on the association between nature and service perception. J. Environ. Psychol. 91:102113. doi: 10.1016/j.jenvp.2023.102113

[ref9004] Lempert K. M. Johnson E. Phelps E. A. (2016). Emotional arousal predicts intertemporal choice. Emotion. 16, 647–656. doi: 10.1037/emo0000168 26882337 PMC4980249

[ref40] Li X. Hsee C. K. O’Brien E. (2023). “It could be better” can make it worse: when and why people mistakenly communicate upward counterfactual information. J. Mark. Res. 60, 219–236. doi: 10.1177/00222437221112312

[ref38] Li Y. Eastman J. (2023). As cute as a button: the effect of size on online product cuteness perception. J. Prod. Brand. Manag. 32, 1306–1318. doi: 10.1108/JPBM-11-2022-4212

[ref39] Li Y. J. Haws K. L. Griskevicius V. (2019). Parenting motivation and consumer decision-making. J. Consum. Res. 45, 1117–1137. doi: 10.1093/jcr/ucy038

[ref41] Li Y. Yan D. (2021). Cuteness inspires men’s risk seeking but women’s risk aversion. J. Bus. Res. 126, 239–249. doi: 10.1016/j.jbusres.2020.12.066

[ref42] Lorenz K. (1970). Studies in animal and human behaviour. Cambridge, MA: Harvard University Press.

[ref43] Lucia-Palacios L. Pérez-López R. Polo-Redondo Y. (2018). Can social support alleviate stress while shopping in crowded retail environments? J. Bus. Res. 90, 141–150. doi: 10.1016/j.jbusres.2018.05.018

[ref44] Luo L. Z. Li H. Lee K. (2011). Are children’s faces really more appealing than those of adults? Testing the baby schema hypothesis beyond infancy. J. Exp. Child Psychol. 110, 115–124. doi: 10.1016/j.jecp.2011.04.002, PMID: 21536307 PMC3105163

[ref45] May F. (2017). The effect of future event markers on intertemporal choice is moderated by the reliance on emotions versus reason to make decisions. J. Consum. Res. 44, ucw081–ucw331. doi: 10.1093/jcr/ucw081

[ref46] May F. Monga A. (2014). When time has a will of its own, the powerless don’t have the will to wait: anthropomorphism of time can decrease patience. J. Consum. Res. 40, 924–942. doi: 10.1086/673384

[ref47] Mikulincer M. Florian V. Hirschberger G. (2003). The existential function of close relationships: introducing death into the science of love. Personal. Soc. Psychol. Rev. 7, 20–40. doi: 10.1207/S15327957PSPR0701_2, PMID: 12584055

[ref48] Mogilner C. (2010). The pursuit of happiness: time, money, and social connection. Psychol. Sci. 21, 1348–1354. doi: 10.1177/0956797610380696 20732902

[ref49] Molden D. C. Hui C. M. Scholer A. A. Meier B. P. Noreen E. E. D’Agostino P. R.. (2012). Motivational versus metabolic effects of carbohydrates on self-control. Psychol. Sci. 23, 1137–1144. doi: 10.1177/0956797612439069, PMID: 22972907

[ref50] Nenkov G. Y. Scott M. L. (2014). “So cute I could eat it up”: priming effects of cute products on indulgent consumption. J. Consum. Res. 41, 326–341. doi: 10.1086/676581

[ref51] Nicholas R. F. Gullone E. (2001). Cute and cuddly and a whole lot more? A call for empirical investigation into the therapeutic benefits of human–animal interaction for children. Behav. Change 18, 124–133. doi: 10.1375/bech.18.2.124

[ref52] Pyone J. S. Isen A. M. (2011). Positive affect, intertemporal choice, and levels of thinking: increasing consumers’ willingness to wait. J. Mark. Res. 48, 532–543. doi: 10.1509/jmkr.48.3.532

[ref53] Rick S. I. Cryder C. E. Loewenstein G. (2008). Tightwads and spendthrifts. J. Consum. Res. 34, 767–782. doi: 10.1086/523285

[ref54] Romero M. Craig A. W. Kumar A. (2019). Mapping time: how the spatial representation of time influences intertemporal choices. J. Mark. Res. 56, 620–636. doi: 10.1177/0022243719827967

[ref55] Schnitker S. A. Houltberg B. Dyrness W. Redmond N. (2017). The virtue of patience, spirituality, and suffering: integrating lessons from positive psychology, psychology of religion, and Christian theology. Psychol. Relig. Spir. 9, 264–275. doi: 10.1037/rel0000099

[ref56] Septianto F. Kwon J. (2022). Too cute to be bad? Cute brand logo reduces consumer punishment following brand transgressions. Int. J. Res. Mark. 39, 1108–1126. doi: 10.1016/j.ijresmar.2021.12.006

[ref57] Septianto F. Paramita W. (2021). Cute brand logo enhances favorable brand attitude: the moderating role of hope. J. Retail. Consum. Serv. 63:102734. doi: 10.1016/j.jretconser.2021.102734

[ref58] Shaddy F. Lee L. (2020). Price promotions cause impatience. J. Mark. Res. 57, 118–133. doi: 10.1177/0022243719871946

[ref59] Sherman G. D. Haidt J. (2011). Cuteness and disgust: the humanizing and dehumanizing effects of emotion. Emot. Rev. 3, 245–251. doi: 10.1177/1754073911402396

[ref60] Sherman G. D. Haidt J. Coan J. A. (2009). Viewing cute images increases behavioral carefulness. Emotion 9, 282–286. doi: 10.1037/a0014904, PMID: 19348541

[ref61] Shin J. Mattila A. S. (2021). Aww effect: engaging consumers in “non-cute” prosocial initiatives with cuteness. J. Bus. Res. 126, 209–220. doi: 10.1016/j.jbusres.2020.11.046

[ref62] Siddiqui R. A. Monga A. Buechel E. C. (2018). When intertemporal rewards are hedonic, larger units of wait time boost patience. J. Consum. Psychol. 28, 612–628. doi: 10.1002/jcpy.1019

[ref63] Song H. Gao R. Zhang Q. Li Y. (2023). The nonlinear effect of time pressure on innovation performance: new insights from a meta-analysis and an empirical study. Front. Psychol. 13:1049174. doi: 10.3389/fpsyg.2022.1049174, PMID: 36698585 PMC9868248

[ref64] Song X. Huang F. Li X. (2017). The effect of embarrassment on preferences for brand conspicuousness: the roles of self-esteem and self-brand connection. J. Consum. Psychol. 27, 69–83. doi: 10.1016/j.jcps.2016.05.001

[ref65] Steinnes K. K. Blomster J. K. Seibt B. Zickfeld J. H. Fiske A. P. (2019). Too cute for words: cuteness evokes the heartwarming emotion of Kama muta. Front. Psychol. 10:387. doi: 10.3389/fpsyg.2019.00387, PMID: 30881329 PMC6405428

[ref66] Sun H.-Y. Ma J.-T. Zhou L. Jiang C.-M. Li S. (2022). Waiting is painful: the impact of anticipated dread on negative discounting in the loss domain. Judgm. Decis. Mak. 17, 1353–1378. doi: 10.1017/S1930297500009451

[ref67] Tai K. Zheng X. Narayanan J. (2011). Touching a teddy bear mitigates negative effects of social exclusion to increase prosocial behavior. Soc. Psychol. Personal. Sci. 2, 618–626. doi: 10.1177/1948550611404707

[ref68] Thompson D. V. Hamilton R. W. Banerji I. (2020). The effect of childhood socioeconomic status on patience. Organ. Behav. Hum. Decis. Process. 157, 85–102. doi: 10.1016/j.obhdp.2020.01.004

[ref69] Waitwhile. (2018). Consumer survey: the state of waiting in line. Available at: https://waitwhile.com/blog/consumer-survey-waiting-in-line-2023/.

[ref71] Wang T. Mukhopadhyay A. Patrick V. M. (2017). Getting consumers to recycle NOW! When and why cuteness appeals influence prosocial and sustainable behavior. J. Public Policy Mark. 36, 269–283. doi: 10.1509/jppm.16.089

[ref72] Witowska J. Schmidt S. Wittmann M. (2020). What happens while waiting? How self-regulation affects boredom and subjective time during a real waiting situation. Acta Psychol. 205:103061. doi: 10.1016/j.actpsy.2020.103061, PMID: 32203734

[ref73] Xu Q. Zhou Y. Ye M. Zhou X. (2015). Perceived social support reduces the pain of spending money. J. Consum. Psychol. 25, 219–230. doi: 10.1016/j.jcps.2014.11.004

[ref74] Yang C. Zhao M. Xie C. Li J. (2022). The influence of infant schema cues on donation intention in charity promotion. Front. Psychol. 13:869458. doi: 10.3389/fpsyg.2022.869458, PMID: 35910966 PMC9326485

[ref75] Zhang Y. Shrum L. J. (2009). The influence of self-construal on impulsive consumption. J. Consum. Res. 35, 838–850. doi: 10.1086/593687

[ref9005] Zhong C. B. DeVoe S. E. (2010). You are how you eat: fast food and impatience. Psychol. Sci. 21, 619–622. doi: 10.1177/0956797610366090 20483836

[ref76] Zhou X. Gao D.-G. (2008). Social support and money as pain management mechanisms. Psychol. Inq. 19, 127–144. doi: 10.1080/10478400802587679

[ref77] Zimet G. D. Powell S. S. Farley G. K. Werkman S. Berkoff K. A. (1990). Psychometric characteristics of the multidimensional scale of perceived social support. J. Pers. Assess. 55, 610–617. doi: 10.1080/00223891.1990.9674095 2280326

