# Supplementary material for: So cute, I could wait: the effect of cuteness on consumer patience
Source: Front Psychol. 2024 May 13;15:1380505. doi: 10.3389/fpsyg.2024.1380505 (PMC11129765; doi:10.3389/fpsyg.2024.1380505)
Supplement: Supplementary file 1 [file Data_Sheet_1.docx]

Supplementary Material

# Appendix A. Cookie designs (Study 1)

| 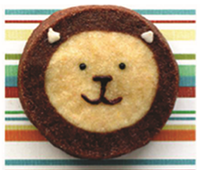  Cute condition | 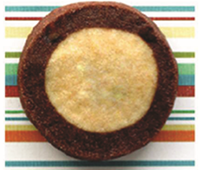  Control condition |
| --- | --- |

# Appendix B. Cuteness stimuli (Studies 2, 3, and 4)

Cute condition: lion cub

| 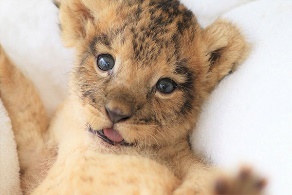 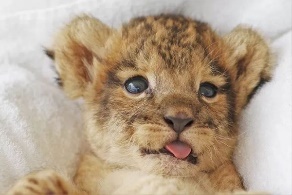 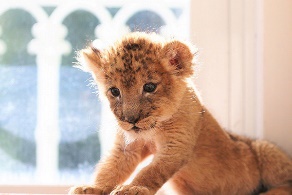 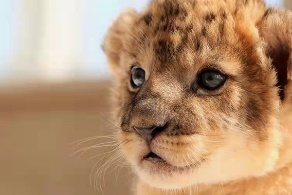 |
| --- |

Control condition: adult lion

| 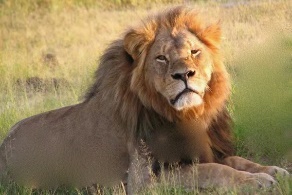 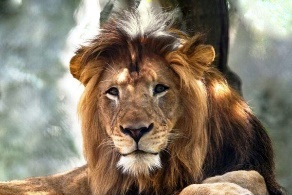 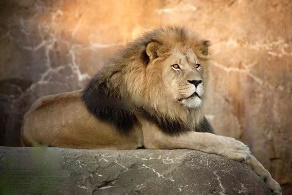 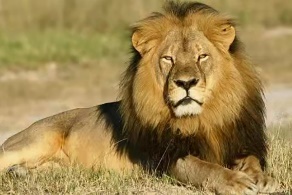 |
| --- |

# Appendix C. Waiting scenario: Restaurant (Study 2)


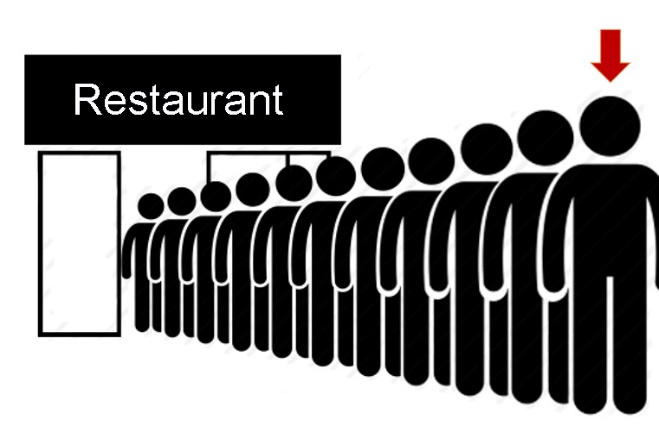


# Appendix D. Waiting scenario: Bus stop (Study 3)


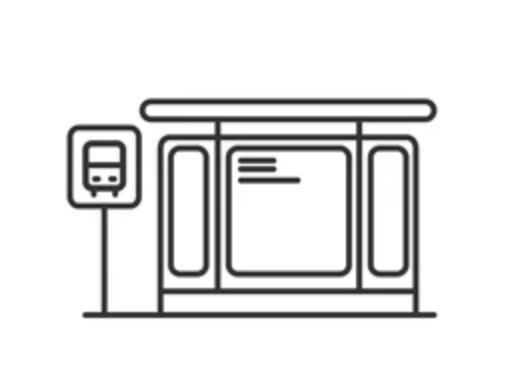


# Appendix E. Real waiting scenario (Study 4)


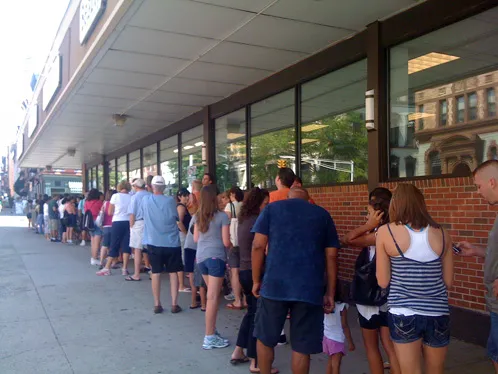


# Appendix F. Queue ticket (Study 5)

| 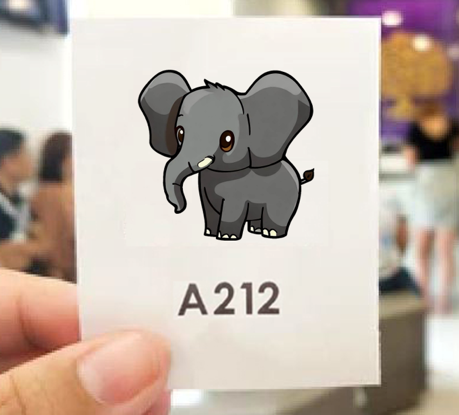  Cute condition | 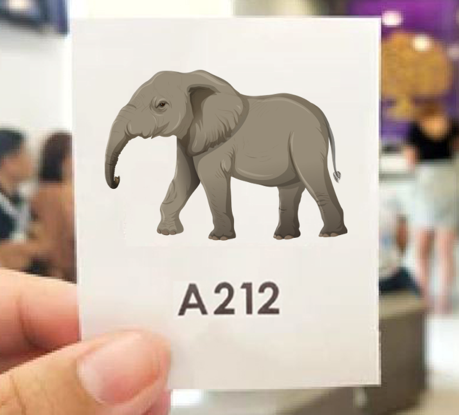  Control condition |
| --- | --- |
